# Lung perfusion changes in COVID-19 pneumonia: a dual energy computerized tomography study

**DOI:** 10.1259/bjr.20201380

**Published:** 2021-08-18

**Authors:** Sonay Aydin, Mecit Kantarci, Erdal Karavas, Edhem Unver, Seven Yalcin, Fahri Aydin

**Affiliations:** 1 Department of Radiology, Erzincan University, Erzincan, Turkey; 2 Department of Radiology, Atatürk University, Erzurum, Turkey; 3 Department of Chest Disease, Erzincan University, Erzincan, Turkey

## Abstract

**Objective:**

There is limited and contradictory information about pulmonary perfusion changes detected with dual energy computed tomography (DECT) in COVID-19 cases. The purpose of this study was to define lung perfusion changes in COVID-19 cases with DECT, as well as to reveal any possible links between perfusion changes and laboratory findings.

**Methods:**

Patients who had a positive RT-PCR for SARS-CoV-2 and a contrast-enhanced chest DECT examination were included in the study. The pattern and severity of perfusion deficits were evaluated, as well as the relationships between perfusion deficit severity and laboratory results and CT severity ratings. The paired t-test, Wilcoxon test, and Student’s t-test were used to examine the changes in variables and perfusion deficits. *p* < 0.05 was regarded as statistically significant.

**Results:**

Study population consisted of 40 patients. Mean age was 60.73 ± 14.73 years. All of the patients had perfusion deficits at DECT images. Mean perfusion deficit severity score of the population was 8.45 ± 4.66 (min.-max, 1–19). In 24 patients (60%), perfusion deficits and parenchymal lesions matched completely. In 15 patients (37.5%), there was partial match. D dimer, CRP levels, CT severity score, and perfusion deficit severity score all had a positive correlation

**Conclusions:**

Perfusion deficits are seen not only in opacification areas but also in parenchyma of normal appearance. The CT severity score, CRP, D-dimer, and SpO2 levels of the patients were determined to be related with perfusion deficit severity.

**Advances in knowledge:**

Findings of the current study may confirm the presence of micro-thrombosis in COVID-19 pneumonia.

## Introduction

Coronavirus disease of 2019 (COVID-19) is known to cause systemic coagulation disorders and microangiopathy. D-dimer and fibrinogen degradation product levels can be elevated in COVID-19 cases, and these levels are associated with a poor prognosis. Vasculopathy is more prominent in lung vessels.^1–3^ Pulmonary embolism (PE) is seen at increased rates in COVID-19 (20.6%–40%), and the presence of PE is related with a severe disease course.^1,4^ In addition, postmortem studies have revealed pulmonary microvascular thrombosis in COVID-19 cases.^5,6^ Severe hypoxemia has frequently been found in COVID-19 disease, regardless of the presence of PE. There are considered to be three mechanisms that can lead to hypoxemia; (a) changes to hypoxic pulmonary vasoconstriction, (b) thrombosis mediated perfusion defects, and (c) ventilation-perfusion mismatching in the normal parenchyma.^7^

Dual-energy CT (DECT) can provide sufficient diagnostic information about PE presence and lung perfusion in a single session. There is also acceptable agreement between DECT perfusion and scintigraphy.^8,9^

Information about pulmonary perfusion changes detected with DECT in COVID-19 cases is limited and contradictory. Some of the publications are case presentations^9,10^ and there is no consensus on perfusion deficit pattern and the matching of perfusion deficits and parenchymal lesions of COVID-19.^11–13^ DECT could help to confirm the mechanism of hypoxemia in COVID-19 by defining the presence and extent of pulmonary perfusion deficits.

The main aim of this study was to describe lung perfusion changes in COVID-19 cases with DECT. A secondary aim was to reveal possible relationships between perfusion changes and imaging/laboratory findings.

## Methods and materials

Approval for this retrospective study was granted by the Institutional Review Board. Informed consent was waived because of the retrospective design.

The study included patients with a positive reverse transcription polymerase chain reaction (RT-PCR) result obtained from a nasopharyngeal swab for severe acute respiratory syndrome coronavirus 2 (SARS–CoV–2) (The swabs were taken before the hospital admission), a contrast-enhanced chest DECT examination, and a chest CT scan prior to the DECT (within 24 h of the first positive RT-PCR result). Patients were excluded from the study if they had low-quality DECT scans (seven patients). Low-quality DECT scans were inappropriate for perfusion deficit determination, since they contained so much artifacts resulting from patients’ respiratory movements, contrast-enhancement was not adequate, as a result parenchymal perfusion could not be evaluated sufficiently, and extensive cardiac movement artifacts presence making the evaluation of middle segments of the lungs impossible. Included DECT examinations were performed between April and October 2020. The study population consisted of 40 patients.

In the current study’s hospital, DECT is generally preferred over standard CT pulmonary angiography for inpatients and intensive care unit patients with worsening clinical conditions. DECT scans are carried out with the agreement of the demanding clinician and the radiologist. DECT examinations were performed for following reasons according to medical records: worsening dyspnea (15 patients), decrease in oxygen saturation levels (11 patients), sudden onset chest pain (three patients), worsening chest pain (nine patients), increase in D dimer and CRP levels (three patients), no record to explain the reason of DECT scan (seven patients).

Age and gender data were collected. Oxygen saturation (SpO2), urea, creatinine, albumin, alanine aminotransferase (ALT), aspartate aminotransferase (AST), lactate dehydrogenase (LDH), total protein, D dimer, ferritin, white blood cell (WBC), hemoglobin (Hb), platelet (Plt), lymphocyte (Lct), neutrophil (Neu), fibrinogen, procalcitonin, C-reactive protein (CRP), troponin, and sedimentation values were recorded both at the time of first COVID-19 diagnosis (on the same day as the first positive RT-PCR result) and at the time the DECT scans were obtained. Laboratory results acquired at the time of the DECT scan (on either the same or the following day). The median interval between the two laboratory results was 12 days (10–14 days).

The CT severity scores were calculated using the Pan et al. method^14^ (Table 1). CT severity scores were calculated for both CT scans: when the first positive RT-PCR result was obtained and by using DECT images.

**Table 1.** CT severity and perfusion deficit severity scores

CT severity score	Extent of lesions for each lung lobe
0	0%
1	<5%
2	5–25%
3	26–50%
4	51–75%
5	>75%
**Perfusion deficit severity score**	**Extent of perfusion deficits for each lung lobe**
0	0%
1	<5%
2	5–25%
3	26–50%
4	51–75%
5	>75%

CT, Computed tomography.

Scores were defined for each lobe and the sum of the scores of the lobes constitutes the total lung score.

Total score scale: 0–25.

The DECT images were acquired using a third-generation dual-source CT scanner (Somatom Force, Siemens Healthineers, Germany). Iohexol of 50–60 ml (350 mgI/100 ml) was administered intravenously (rate = 4.0 ml s^−1^) via an antecubital vein followed by a 40-ml saline chaser bolus. Using a bolus-tracking technique, a region of interest (ROI) was placed over the main pulmonary artery, and the acquisition was started when the ROI reached 100 Hounsfield Unit (HU). After scout acquisition, imaging was acquired in the supine position, in a cranio-caudal direction with the following parameters: 90/150 Sn kVp, 60 mAs, and rotation time 0.33 s. Imaging reconstruction was performed in axial, coronal, and sagittal planes with 1.5-mm slice thickness.

**Figure 1.**
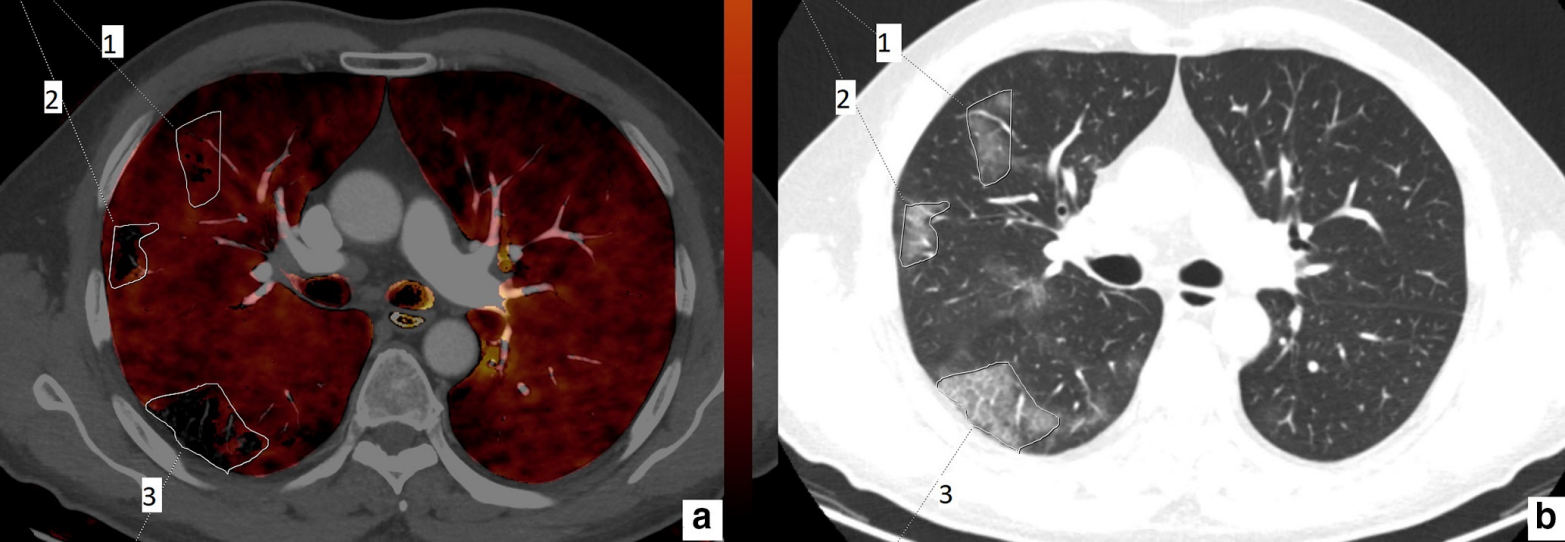
61-year-old male. Axial perfusion blood volume (a) and computed tomography (b) images. In lesion 1, ground glass opacity area is larger than the perfusion deficit. In lesions 2 and 3, the perfusion deficit and ground glass opacity areas are the same.

The DECT images were assessed on a workstation (Syngo.via, Siemens Healthineers, Erlangen, Germany) by two by two chest radiologists with 24 and 13 years' experience blinded to each other’s findings and clinical data. In contradictory cases, the opinion of a third radiologist (8 years of experience) was applied and final decisions were made by consensus. Perfused blood volume (PBV) images and iodine maps were generated using DECT post-processing software (“CT dual energy”,” Lung Analysis”, “Lung PBV”, and “Virtual unenhanced”). PBV images were used to detect the presence and extent of the perfusion deficits (Color map details, Color LUT CT: gray scale, Color LUT overlay: hot body. Window settings: W: 600/200, C:150/100). A perfusion deficit severity score was created (Table 1) according to the extent of the deficits and the scores were calculated for each patient. To define the matching or mismatching between perfusion deficits and parenchymal lesions (ground glass opacification [GGO] or consolidation), simultaneous evaluations were made of PBV images and standard CT images with lung parenchyma windowing. The amount of matching between perfusion deficits and parenchymal lesions was recorded as a percentage. For cases with partial matching, areas of perfusion deficit and parenchymal lesions were calculated via the freehand ROI function, and the matching percentage was defined using these areas (Figure 1). In addition to PBV images, iodine maps were created. The iodine uptake values of the GGOs and consolidations were found by placing ROI circles on the iodine maps. Three ROI circles were placed on the lesions and the mean value of the three measurements was recorded as the final value.

Statistical analysis: Data were analyzed using the Statistical Package for Social Sciences (SPSS) for Windows v.20 software (IBM SPSS Inc., Chicago, IL, USA). Normal distribution of the data was evaluated with the Kolmogorov–Smirnov test. Numerical variables with normal distribution were shown as mean ± standard deviation values, and variables without normal distribution were shown as median (minimum-maximum) values. Categorical variables were stated as percentages. The change in variables from initial diagnosis to the time of the DECT scan was analyzed with the paired t-test and the Wilcoxon test. The Student’s t-test was used to compare the severity of perfusion deficits in patients with increasing and decreasing CT severity scores. Spearman and Pearson correlation analyses were applied to define possible correlations between the perfusion deficit severity score and the parameters evaluated. Categorical correlation analysis (Cohen κ values- ĸ) was used to define interobserver agreement (κ results can be interpreted as follows: values ≤ 0 no agreement and 0.01–0.20 as none to slight, 0.21–0.40 as fair, 0.41–0.60 as moderate, 0.61–0.80 as substantial, and 0.81–1.00 as strong agreement). A two-tailed value of *p* < 0.05 was considered statistically significant.

## Results

Evaluation was made of 18 males (45%) and 22 females (55%) with a mean age of 60.73 ± 14.73 years (22–87 years).

Laboratory parameters and mean CT severity scores at the time of diagnosis and DECT scan can be seen in Table 2. The median interval between a positive RT-PCR result and a DECT scan, as well as between the first CT scan and the DECT scan was 12 days (10–14 days). All the included patients were administered 6000 anti-Xa IU/ 0.6 ml enoxaparin according to the treatment guidelines of the Ministry of Health of Turkey.^15^

**Table 2.** Values of the parameters at initial diagnosis and at the time of DECT scan

Parameters	Initial Diagnosis	DECT scan	*p*-value
**CT severity score**	**5.9 ± 2.85**	**7.95 ± 3.65**	**0.002**
**Oxygen saturation (%**)	**93.27 ± 2.91**	**90.06 ± 5.73**	**0.04**
BUN (mg/dL)	35.38 ± 16.91	34.73 ± 14	0.55
**Creatinine (mg/dL**)	**0.77 (0.54–1.42**)	**0.91 (0.6–1.13**)	**0.04**
**Albumin (g/dl**)	**38.7 ± 3.96**	**34.07 ± 3.35**	**0.001**
**ALT (U/L**)	**34.25 ± 22.94**	**45 ± 28.2**	**0.03**
AST (U/L)	29 (17–127)	27 (9–86)	0.12
LDH (U/L)	275.28 ± 107.73	281.25 ± 142.5	0.24
Total protein (g/dL)	68.27 ± 9.34	65.12 ± 5.29	0.69
**D-dimer (μg/L**)	**771.53 ± 467.91**	**1147.45 ± 2520.11**	**0.001**
**Fibrinogen (mg/dL**)	**314.49 ± 68,07**	**362.39 ± 79.99**	**0.001**
**White blood cell (×10^9^/L**)	**5.45 (3.9–11.8**)	**7.25 (3.1–16.8**)	**0.001**
**Platelet (×10^9^/L**)	**204.17 ± 65.57**	**269.15 ± 99.66**	**0.005**
**Neutrophil count (×10^9^/L**)	**3.45 (2.11–10.24**)	**5.36 (1.65–14.86**)	**0.001**
Lymphocyte count (×10^9^/L) Lymphocyte count %	1.25 (0.5–2.53) 28.1% (11.2–56.9%)	1.15 (0.4–3.3) 25.8% (9–74.2%)	0.47
**Hemoglobin (g/dL**)	**13.43 ± 1.46**	**12.66 ± 1.38**	**0.007**
Ferritin (μg/L)	264.01 ± 233.44	276.57 ± 303.16	0.5
**CRP (mg/dL**)	**27.45 (3.02–167**)	**43.92 (2-132**)	**0.002**

ALT, Alanine amino transferase; AST, Aspartate amino transferase; BUN, Blood urea nitrogen; CRP, C reactive protein; CT, Computed tomography; LDH, Lactate dehydrogenase.

Parameters with normal distribution are shown as mean ± standard deviation, parameters without normal distribution are shown as median (minimum-maximum) values.

Bold indicates statistical significance.

**Figure 2.**
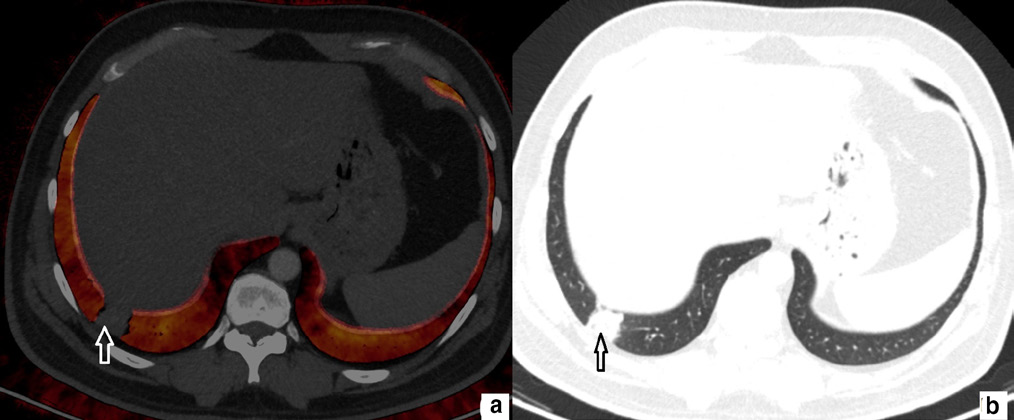
52-year-old female. Axial perfusion blood volume (a) and computed tomography (b) images. Focal consolidation at posterior basal segment (b, arrow), matching perfusion deficit is present on perfusion blood volume image (a, arrow).

**Figure 3.**
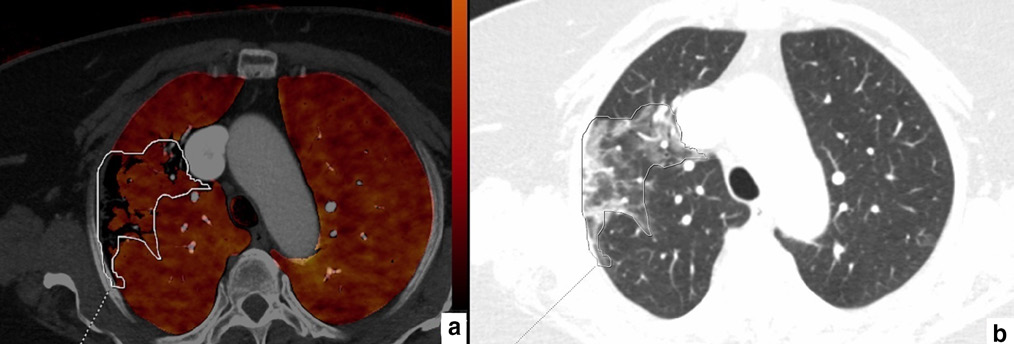
64-year-old female. Axial perfusion blood volume (a) and computed tomography (b) images. In the right upper lobe, a large, heterogeneous area of GGOs and consolidations can be seen (b, marked area). Perfusion deficits are also present in the same area, but they take up less space than GGOs and consolidations (a, marked area).

**Figure 4.**
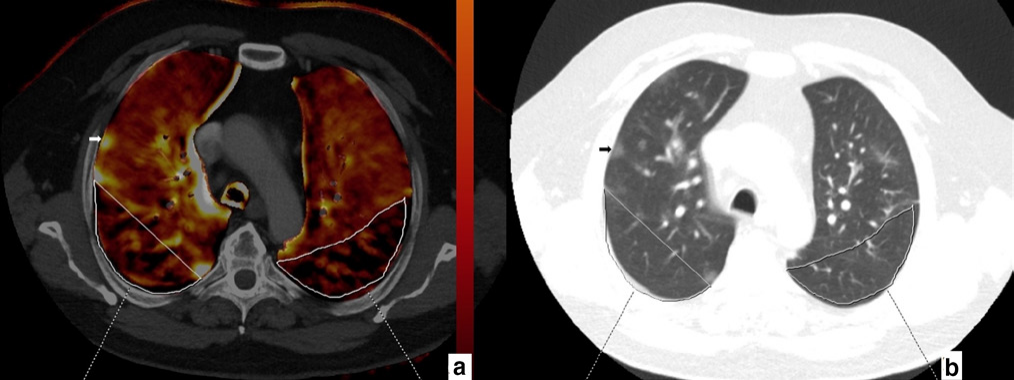
67-year-old male. Axial perfusion blood volume (a) and computed tomography (b) images. Large, heterogeneous perfusion deficit areas are seen in the posterior zones of upper lobes (a, marked areas). No GGO or consolidation is present in the perfusion deficit areas (b, marked areas). Ground glass opacification in the right upper lobe peripheral zone (b, arrow) does not reveal any perfusion deficit (a, arrow).

**Figure 5.**
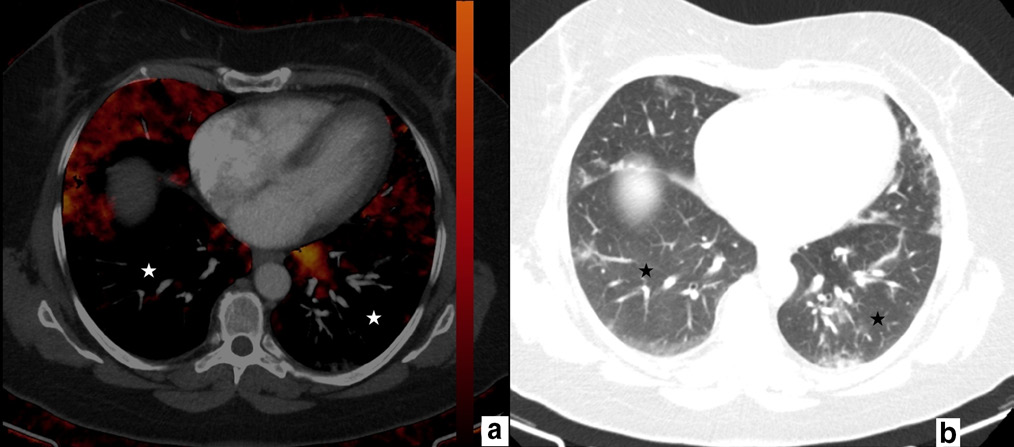
77-year-old male. Axial perfusion blood volume (a) and computed tomography (b) images. Peripherally distributed ground glass opacifications and focal consolidations are present (b), large areas of perfusion deficits in the bilateral lower lobes are present on perfusion blood volume images (a, stars). Perfusion deficit areas occupy a larger area than GGOs and consolidations and mainly appear normal on computed tomography (b, stars).

All of the patients were determined to have perfusion deficits on the DECT images, and no PE was detected on CT pulmonary angiography images. The mean perfusion deficit severity score of the whole study population was 8.45 ± 4.66 (min–max, 1–19). Mean perfusion deficit severity score was higher in patients whose CT severity score increased between the first and the second CT scans (9.92 ± 4.94 vs 6.25 ± 3.23, *p* = 0.01). In 24 patients (60%), perfusion deficits and parenchymal lesions matched completely (Figure 2) and in one patient (2.5%), perfusion deficits and parenchymal lesions did not match at all. In 15 patients (37.5%), there was a partial match between perfusion deficits and parenchymal lesions. Of these patients, parenchymal involvement was larger than perfusion deficits in two patients (2/15, 13.3%) (Figure 3), and in the rest (13/15, 86.6%), perfusion deficits were than parenchymal lesions (Figures 4 and 5). In the whole study population, the mean-matching percentage was 81.37±29.39%. No significant correlation was determined between matching percentage and initial CT severity score, CT severity score at DECT scan, and perfusion deficit severity score (*p* > 0.05).

In mismatching and partially matching patients (16/40), perfusion deficits were generally located at posterior-peripheral areas of both lungs (Figures 4 and 5): perfusion deficits were located predominantly in posterior zones of both lungs in 13 patients (13/16, 81.2%), in middle zones in two patients (2/16, 12.5%), and in anterior zones in one patient (1/16, 6.2%). The deficits were mostly found in the peripheral areas in 14 patients (14/16, 87.5%) and in the central areas in two patients (2/16, 12.5%).

In six patients (15%), no consolidations were observed, only GGOs. The Hounsfield Unit (HU) values of consolidations acquired from iodine uptake maps were higher than those of GGOs (−97.29 ± −91.29 *vs* -565.6 ± 102.3, accordingly).

**Figure 6.**
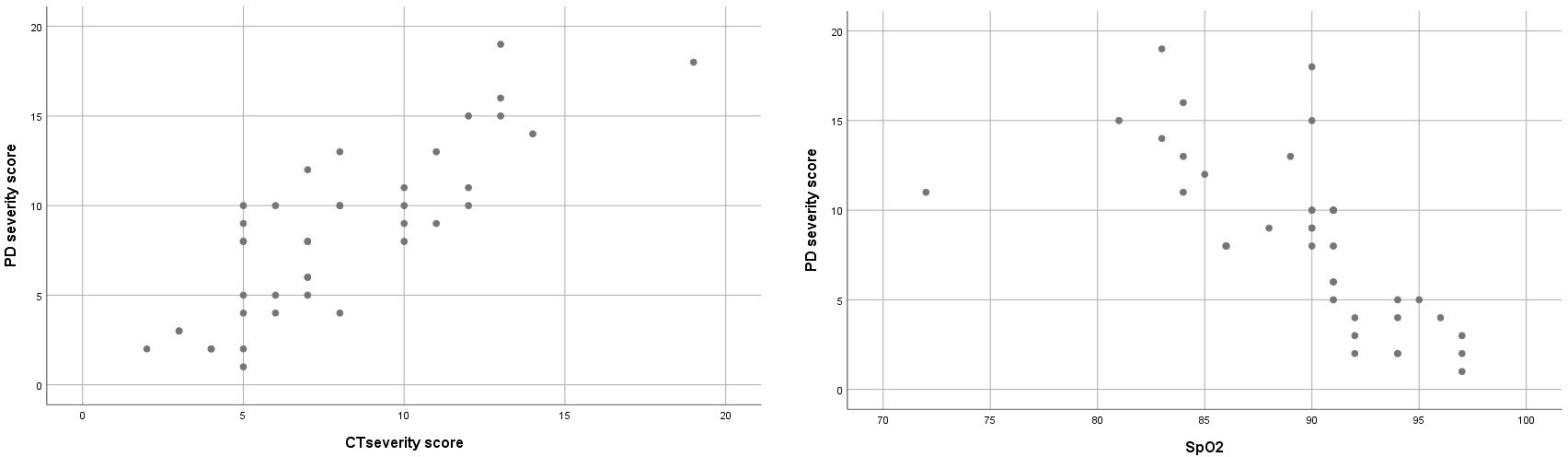
Scatter plots for the correlation of perfusion deficit against CT severity score and Oxygen saturation (PD: Perfusion deficit, Oxygen saturation: Spo2).

The correlation analysis results showed a positive correlation between initial D dimer, CRP levels, CT severity score and perfusion deficit severity score. A negative correlation was determined between initial SpO2 values and perfusion deficit severity score (Table 3 and Figure 6).

**Table 3.** Correlations between perfusion deficit severity score and the parameters

Parameters (at the time of initial diagnosis)	Correlations with perfusion deficit severity score	
	R	*p* value
Age	0.186	0.125
**CT severity score**	**0.338**	**0.033**
**Oxygen saturation**	−**0.752**	**0.001**
BUN	0.097	0.55
Creatinine	0.018	0.91
Albumin	−0.132	0.41
ALT	0.309	0.052
AST	0.415	0.08
LDH	0.012	0.32
Total protein	0.042	0.71
**D-dimer**	**0.412**	**0.015**
Fibrinogen	0.34	0.08
White blood cell	0.04	0.8
Platelet	0.16	0.32
Neutrophil count	0.13	0.39
Lymphocyte count	−0.22	0.15
Hemoglobin	0.08	0.62
Ferritin	0.29	0.15
**CRP**	**0.399**	**0.011**

ALT, Alanine amino transferase; AST, Aspartate amino transferase; BUN, Blood urea nitrogen; CRP, C reactive protein; CT, Computed tomography; LDH, Lactate dehydrogenase.

Bold indicates statistical significance.

37 patients were discharged from the hospital without any noted complications. Following the DECT scan, two patients were transferred to the intensive care unit (ICU) due to acute renal failure and congestive heart failure caused by previous mitral valve prolapse, and were eventually discharged from the hospital. one patient (an 87-year-old male) died after being admitted to the ICU, primarily due to septicemia caused by infection of previously present multiple diabetic wounds.

Interobserver agreement was found to be strong for both the initial and second CT severity scoring (values 0.87 and 0.89, respectively). Interobserver agreement was also high for perfusion deficit severity scoring and the percentage of deficit-parenchymal lesion matching (values 0.84 and 0.82, respectively).

## Discussion

The primary goal of this research was to use DECT to detect lung perfusion deficits in COVID-19 patients and to compare these deficits with CT and laboratory findings. The perfusion properties of COVID-19 patients are heterogeneous. GGOs and consolidations revealed perfusion deficits in the majority of cases, but these deficits could also be seen in parenchyma that appeared normal The occurrence of perfusion deficits was discovered to be related to the severity ratings of the patients' CT scans Acute hypoxemic respiratory failure is a serious complication of COVID-19 pneumonia. According to studies, lung compliance is preserved in hypoxemic respiratory failure cases, indicating that the cause is not always alveolar damage. As a result, microvascular thrombosis is the most likely cause of severe hypoxemia.^16,17^ Ventilation/perfusion anomalies have been identified in COVID-19 cases without PE, lending support to the vascular basis of hypoxemia.^18,19^

DECT has been shown to be effective in detecting small PEs that conventional CT angiography cannot detect, and it is also useful for detecting parenchymal perfusion deficits.^20^ Despite a large number of publications on COVID-19 pneumonia and its associated CT properties, information about DECT findings and relevant perfusion deficits is limited and partially contradictory.

Perfusion deficits were present in all included patients and none of them had PEs to cause those deficits. Similarly, Lang et al^10^ and Afat et al^12^ reported perfusion deficits in a population with COVID-19 diagnosis and without PEs. Idilman et al^11^ and Grillet et al^21^ studied a more heterogeneous population of patients with and without PEs, and came to the same conclusion that perfusion deficits could be seen in COVID-19 cases without the presence of PE. Pulmonary microvascular damage and occlusion caused by endothelitis has been previously defined.^5,6^ This microvascular damage is most likely to be the main cause of perfusion deficits.

DECT examinations were performed during enoxaparin treatment. Previous DECT studies have not provided any information about the effect of anticoagulant treatment on perfusion deficits. In a recent study, the definition of “pulmonary *in situ* thrombosis” was offered for the micro thrombosis of COVID-19 pneumonia and it was suggested that thrombosis prevention should rely not only on anticoagulant therapy but also on antiplatelet agents.^22^ Aspirin use has recently been linked to better outcomes in COVID-19 hospitalized patients.^23^ When the perfusion deficits in patients taking enoxaparin are considered, the current study results can be interpreted as confirmation of the need for an additional antiplatelet agent. Furthermore, in light of the current study’s findings, the dosage of enoxaparin treatment may be reconsidered. In previous studies, it has been emphasized that enoxaparin treatment significantly reduced PE presence.^24,25^ We cannot detect any PE cases within our study population. Absence of PE cases can be attributed to enoxaparin treatment.

In the previous studies, perfusion deficits of COVID-19 were defined as heterogeneous.^12,19,21^ Lang et al^10^ reported that perfusion deficits were seen in GGOs and consolidations. On the other hand, one recent study reported that perfusion deficits did not overlap with these lesions.^11^ In line with most of the previous studies, we found that matching of perfusion deficits and GGOs and consolidations was heterogeneous. Generally, there is a match between perfusion deficits and GGOs/consolidations, but perfusion deficits can be seen in normally appearing areas, too. Patients with COVID-19 were shown to suffer from microvascular damage and the vasoconstriction.^26^ These effects might cause the perfusion deficits in normally appearing areas. Perfusion deficits can be encountered in GGO and consolidation areas as a result of damaged ventilation and perfusion ratio. These mechanism could explain the heterogeneous perfusion pattern seen in COVID-19 pneumonia.

Time of acquisition of the DECT can be an alternative explanation for the heterogeneous perfusion deficit pattern and the differences between previous studies. In our population, DECT scans were performed after 10–14 days from the positive RT-PCR scan. Similarly, other previous studies were also performed in hospitalized patients and in the acute stage of COVID-19 pneumonia.^11,12,27^ However, there is no standardized time for DECT scans as of yet. Further research with standardized timing for disease progression or focusing on chronic stage perfusion deficits can provide valuable information. Perfusion deficits were most frequently found in the posterior-peripheral lung areas when not associated with parenchymal opacities. Perfusion deficits may continue to follow a distribution that overlaps with the expected distribution of COVID-19 pneumonia opacities..^28,29^ The similarity in preferred distribution may confirm the veracity of the data presented and can be used as an indicator for the distribution of microvascular damage and vasoconstriction in COVID-19 pneumonia. Furthermore, pulmonary vessel dilatation occurs in COVID-19 pneumonia, not only within lung opacities but also outside of parenchymal opacities and occasionally in the subpleural lung,^29^ which is consistent with the distribution of perfusion deficits in the current study. We were unable to assess the possible links between enlarged vessel presence and perfusion deficits due to the small population size. Additional research with larger populations may reveal associations.

Iodine uptake values tend to increase with the increasing opacification of the parenchyma.^11,21^ The findings of the current study are consistent with the literature in that consolidations have higher iodine uptake values than GGOs. It can be concluded that in the early phase of the disease (GGOs dominant phase) inflammation is at a more limited level, and with the increase of the density of parenchymal lesions (consolidations), the inflammation and contrast enhancement also increases. In some cases, reflex vasoconstriction occurs only after that because of impaired ventilation/perfusion function, and consequently, perfusion deficits occur in lung opacification areas.

Previous COVID-19 pneumonia DECT studies have generally concentrated on the presence and pattern of perfusion deficits.^10,12,21^ There is only one study on the relationships between perfusion deficits and clinical/laboratory findings, in which CRP, D-dimer, troponin, and ferritin levels are linked to the presence of perfusion deficits.^11^ Similarly to those findings, CRP and D-dimer levels were found to be positively correlated with the severity of perfusion deficit in the current study. Some other markers were also seen to increase during the disease course, such as WBC, platelet, creatinine, and ferritin levels. However, no significant correlation was determined between these parameters and the perfusion deficit severity scores. Hence, it was concluded that the severity of perfusion deficits is primarily related with CRP and D-dimer values. The relationships of CRP and D-dimer can be considered to confirm that the pathological base of perfusion deficits involves microvascular thrombosis/injury and inflammation. In addition, SpO2 values were found to be negatively correlated with perfusion deficit scores. As previously mentioned, perfusion deficits and related ventilation/perfusion anomalies can cause hypoxemia.^19^ The relationship with SpO2 values supports the reliability of perfusion deficit severity findings and confirms the clinical consistency of DECT findings.

CT severity scoring systems were found to be a good predictor of COVID-19 prognosis.^30,31^ As far as we know, no previous research has been conducted to investigate the relationships between CT severity scoring and perfusion deficits. According to our results, CT severity scores of the patients are positively correlated with the severity of their perfusion deficit. As previously stated, in addition to being a good predictor of prognosis, CT severity scoring can also be an effective indicator of future perfusion deficit presence.

Previous studies reported good to excellent interobserver agreement for CT severity scoring.^32,33^ In one study, interobserver agreement for perfusion deficits on lung DECT images of COVID-19 cases was evaluated, and the level of agreement was excellent.^12^ In line with the literature, interobserver agreement in the current study for both CT and perfusion deficit severity scoring was at a strong level (ĸ values > 0.8). This validates the reproducibility and dependability of the severity scoring systems employed.

This study has some limitations which should be considered. Although this retrospective study exceeded the population sizes of previous studies, the results may be seen to be different with further prospective studies with larger populations. There were no patients without perfusion deficits in the current study population, so no cut-off value could be calculated to predict the presence of perfusion deficit. Follow-up DECT scans were not available, so it was not possible to provide information about the impact of perfusion deficits on patient prognosis, and also the change of perfusion deficits over time and during recovery. As a result of the retrospective nature, data about the clinical conditions of the patients is limited, we could not correlate perfusion deficit presence/severity with clinical data or outcome. We did not have enough information about the patients’ symptoms prior to the RT-PCR results or the first CT scan, as a result we could not correlate these information with perfusion deficit presence. Even though the presence of perfusion deficits was confirmed with the consensus of at least two radiologists, some abnormalities in the PBV maps could still be related to technical issues. The addition of a control group could be able to overcome these limitations; however, we could not find enough patients to constitute such a control group. In most cases, a more experienced observer resolves a contentious assessment; however, in the current study, a less experienced radiologist was included. Even though the interobserver agreement data were strong, this situation could lead to a limitation. We were unable to detect a statistically significant number of different CT signs in our study population other than ground glass opacities and consolidations, so we could only evaluate the relationships between CT severity and perfusion deficits. Further research with larger populations may shed light on the utility of various CT signs in predicting the presence of a perfusion deficit.

In conclusion, the results of this study demonstrated that lung perfusion deficits in COVID-19 cases can be revealed and evaluated with DECT scanning. Perfusion deficits are seen not only in opacification areas but also in parenchyma of normal appearance. The CT severity score, CRP, D-dimer, and SpO2 levels of the patients were found to be related with perfusion deficit severity. The findings of this study may confirm the presence of micro-thrombosis in COVID-19 pneumonia cases and influence patient management especially in respect of antithrombotic treatment.

## References

[1] Middeldorp S, Coppens M, van Haaps TF, Foppen M, Vlaar AP, Müller MCA, et al. Incidence of venous thromboembolism in hospitalized patients with COVID-19. J Thromb Haemost 2020; 18: 1995–2002. doi: 10.1111/jth.14888 32369666 PMC7497052

[2] Fogarty H, Townsend L, Ni Cheallaigh C, Bergin C, Martin-Loeches I, Browne P, et al. COVID19 coagulopathy in Caucasian patients. Br J Haematol 2020; 189: 1044–9. doi: 10.1111/bjh.16749 32330308 PMC7264579

[3] Ciceri F, Beretta L, Scandroglio AM, Colombo S, Landoni G, Ruggeri A, et al. Microvascular COVID-19 lung vessels obstructive thromboinflammatory syndrome (MicroCLOTS): an atypical acute respiratory distress syndrome working hypothesis. Crit Care Resusc 2020; 22: 95-97. 32294809 10.51893/2020.2.pov2 PMC10692450

[4] Léonard-Lorant I, Delabranche X, Séverac F, Helms J, Pauzet C, Collange O, et al. Acute pulmonary embolism in patients with COVID-19 at CT angiography and relationship to D-dimer levels. Radiology 2020; 296: E189–91. doi: 10.1148/radiol.2020201561 32324102 PMC7233397

[5] Varga Z, Flammer AJ, Steiger P, Haberecker M, Andermatt R, Zinkernagel AS, et al. Endothelial cell infection and endotheliitis in COVID-19. The Lancet 2020; 395: 1417–8. doi: 10.1016/S0140-6736(20)30937-5 PMC7172722 32325026

[6] Ackermann M, Verleden SE, Kuehnel M, Haverich A, Welte T, Laenger F, et al. Pulmonary vascular endothelialitis, thrombosis, and angiogenesis in Covid-19. New England Journal of Medicine 2020; 383: 120–8. doi: 10.1056/NEJMoa2015432 32437596 PMC7412750

[7] Herrmann J, Mori V, Bates JHT, Suki B. Modeling lung perfusion abnormalities to explain early COVID-19 hypoxemia. Nat Commun 2020; 11: 1–9. doi: 10.1038/s41467-020-18672-6 32985528 PMC7522238

[8] Fuld MK, Halaweish AF, Haynes SE, Divekar AA, Guo J, Hoffman EA. Pulmonary perfused blood volume with dual-energy CT as surrogate for pulmonary perfusion assessed with dynamic multidetector CT. Radiology 2013; 267: 747–56. doi: 10.1148/radiol.12112789 23192773 PMC3662901

[9] Masy M, Giordano J, Petyt G, Hossein-Foucher C, Duhamel A, Kyheng M, et al. Dual-Energy CT (DECT) lung perfusion in pulmonary hypertension: concordance rate with V/Q scintigraphy in diagnosing chronic thromboembolic pulmonary hypertension (CTEPH). Eur Radiol 2018; 28: 5100–10. doi: 10.1007/s00330-018-5467-2 29846802

[10] Lang M, Som A, Mendoza DP, Flores EJ, Reid N, Carey D, et al. Hypoxaemia related to COVID-19: vascular and perfusion abnormalities on dual-energy CT. Lancet Infect Dis 2020; 20: 1365–6. doi: 10.1016/S1473-3099(20)30367-4 32359410 PMC7252023

[11] Idilman IS, Telli Dizman G, Ardali Duzgun S, Irmak I, Karcaaltincaba M, Inkaya AC, et al. Lung and kidney perfusion deficits diagnosed by dual-energy computed tomography in patients with COVID-19-related systemic microangiopathy. Eur Radiol 2021; 31: 1090–9. doi: 10.1007/s00330-020-07155-3 32860146 PMC7455509

[12] Afat S, Othman AE, Nikolaou K, Gassenmaier S. Dual-Energy computed tomography of the lung in COVID-19 patients: mismatch of perfusion defects and pulmonary opacities. Diagnostics 2020; 10: 870. doi: 10.3390/diagnostics10110870 33114478 PMC7693945

[13] Patel BV, Arachchillage DJ, Ridge CA, Bianchi P, Doyle JF, Garfield B, et al. Pulmonary angiopathy in severe COVID-19: physiologic, imaging, and hematologic observations. Am J Respir Crit Care Med 2020; 202: 690–9. doi: 10.1164/rccm.202004-1412OC 32667207 PMC7462405

[14] Pan F, Ye T, Sun P, Gui S, Liang B, Li L. Time course of lung changes on chest CT during recovery from 2019 novel coronavirus (COVID-19) pneumonia. Radiology 2020; 295: 715–21. 32053470 10.1148/radiol.2020200370 PMC7233367

[15] Bakanlığı TS. COVID-19 (SARS-CoV-2 Enefksiyonu) Erişkin Hasta Tedavisi. 2020. Available from: https://covid19.saglik.gov.tr/

[16] Chen T, Wu D, Chen H, Yan W, Yang D, Chen G, et al. Clinical characteristics of 113 deceased patients with coronavirus disease 2019: retrospective study. BMJ 2020; 368: m1091. doi: 10.1136/bmj.m1091 32217556 PMC7190011

[17] Klok FA, Kruip MJHA, van der Meer NJM, Arbous MS, Gommers DAMPJ, Kant KM, et al. Incidence of thrombotic complications in critically ill ICU patients with COVID-19. Thromb Res 2020; 191: 145–7. doi: 10.1016/j.thromres.2020.04.013 32291094 PMC7146714

[18] Fielding PA, Morley NCD, Bradley KM. Beware COVID-19 on VQ scans (ventilation/perfusion scintigraphy. QJM: An International Journal of Medicine 2020; 113: 892–3. doi: 10.1093/qjmed/hcaa274 32976600 PMC7543600

[19] Santamarina MG, Boisier D, Contreras R, Baque M, Volpacchio M, Beddings I. COVID-19: a hypothesis regarding the ventilation-perfusion mismatch. Crit Care 2020; 24: 395. doi: 10.1186/s13054-020-03125-9 32631389 PMC7338110

[20] Weidman EK, Plodkowski AJ, Halpenny DF, Hayes SA, Perez-Johnston R, Zheng J, et al. Dual-Energy CT angiography for detection of pulmonary emboli: incremental benefit of iodine maps. Radiology 2018; 289: 546–53. doi: 10.1148/radiol.2018180594 30204073 PMC6209063

[21] Grillet F, Busse-Coté A, Calame P, Behr J, Delabrousse E, Aubry S. COVID-19 pneumonia: microvascular disease revealed on pulmonary dual-energy computed tomography angiography. Quant Imaging Med Surg 2020; 10: 1852–62. doi: 10.21037/qims-20-708 32879862 PMC7417764

[22] Thachil J, Srivastava A. Maintaining hemostasis and preventing thrombosis in COVID-19—Part I: SARS-2 Coronavirus–Associated hemostatic lung abnormality in COVID-19: is it pulmonary thrombosis or pulmonary embolism? Semin Thromb Hemost 2020; 46: 777. 32396963 10.1055/s-0040-1712155 PMC7645824

[23] Chow JH, Khanna AK, Kethireddy S, Yamane D, Levine A, Jackson AM, et al. Aspirin use is associated with decreased mechanical ventilation, intensive care unit admission, and in-hospital mortality in hospitalized patients with coronavirus disease 2019. Anesth Analg 2021; 132: 930–41. doi: 10.1213/ANE.0000000000005292 33093359

[24] Scarduelli C, Inglese F, Beccaria M, Speafico F, Garuti M, Pecoriello A, et al. Pulmonary embolism in patients with severe COVID-19 treated with intermediate- to full-dose enoxaparin: a retrospective study. Monaldi Arch Chest Dis 2021. doi: 10.4081/monaldi.2021.1758 33728885

[25] Scarduelli C, Inglese F, Beccaria M, Speafico F, Garuti M, Pecoriello A, et al. Pulmonary embolism in patients with severe COVID-19 treated with intermediate- to full-dose enoxaparin: a retrospective study. Monaldi Arch Chest Dis 2021;16 Mar 2021. doi: 10.4081/monaldi.2021.1758 33728885

[26] Liu Y, Yang Y, Zhang C, Huang F, Wang F, Yuan J, et al. Clinical and biochemical indexes from 2019-nCoV infected patients linked to viral loads and lung injury. Sci China Life Sci 2020; 63: 364–74. doi: 10.1007/s11427-020-1643-8 32048163 PMC7088566

[27] Le Berre A, Boeken T, Caramella C, Afonso D, Nhy C, Saccenti L, et al. Dual-Energy CT angiography reveals high prevalence of perfusion defects unrelated to pulmonary embolism in COVID-19 lesions. Insights Imaging 2021; 12: 24. doi: 10.1186/s13244-021-00972-0 33595746 PMC7887542

[28] Salehi S, Abedi A, Balakrishnan S, Gholamrezanezhad A. Coronavirus disease 2019 (COVID-19): a systematic review of imaging findings in 919 patients. AJR Am J Roentgenol 2020; 215: 87–93. doi: 10.2214/AJR.20.23034 32174129

[29] Lang M, Som A, Carey D, Reid N, Mendoza DP, Flores EJ, et al. Pulmonary vascular manifestations of COVID-19 pneumonia. Radiol Cardiothorac Imaging 2020; 2: e200277: e200277. doi: 10.1148/ryct.2020200277 34036264 PMC7307217

[30] Pan F, Ye T, Sun P. Time course of lung changes on chest CT during recovery from 2019 novel coronavirus (COVID-19) pneumonia. Radiology 2020; 295: 715–21. 32053470 10.1148/radiol.2020200370 PMC7233367

[31] Francone M, Iafrate F, Masci GM, Coco S, Cilia F, Manganaro L, et al. Chest CT score in COVID-19 patients: correlation with disease severity and short-term prognosis. Eur Radiol 2020; 30: 6808–17. doi: 10.1007/s00330-020-07033-y 32623505 PMC7334627

[32] Yang R, Li X, Liu H, Zhen Y, Zhang X, Xiong Q, et al. Chest CT severity score: an imaging tool for assessing severe COVID-19. Radiol Cardiothorac Imaging 2020; 2: e200047. doi: 10.1148/ryct.2020200047 33778560 PMC7233443

[33] Ufuk F, Demirci M, Uğurlu E, Çetin N, Yiğit N, Sarı T, et al. Evaluation of disease severity with quantitative chest CT in COVID-19 patients. Diagnostic and Interventional Radiology 2020; 27: 164–71. doi: 10.5152/dir.2020.20281 PMC7963378 33044173

